# Fast and Specific Peroxygenase Reactions Catalyzed by Fungal Mono-Copper Enzymes

**DOI:** 10.1021/acs.biochem.1c00407

**Published:** 2021-11-05

**Authors:** Lukas Rieder, Anton A. Stepnov, Morten Sørlie, Vincent G.H. Eijsink

**Affiliations:** Faculty of Chemistry, Biotechnology, and Food Sciences, Norwegian University of Life Sciences (NMBU), P.O. Box 5003, NO, 1432 Ås, Norway

## Abstract

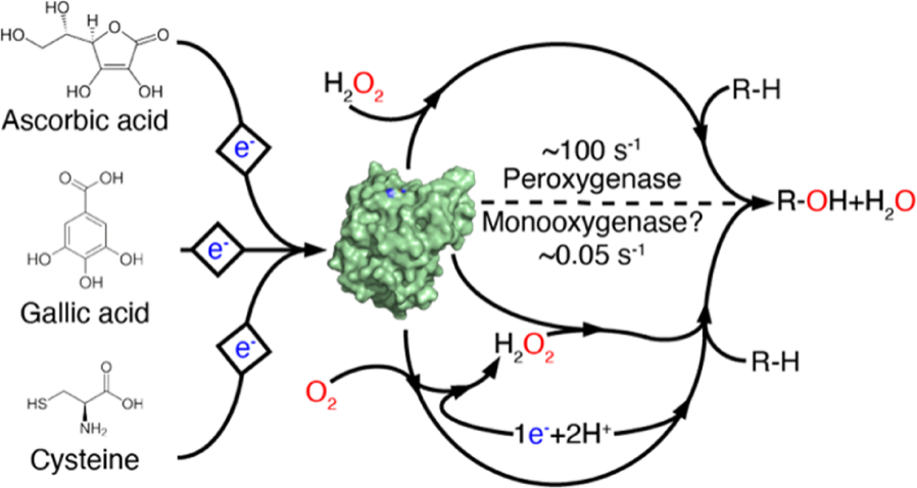

The copper-dependent lytic polysaccharide monooxygenases (LPMOs) are receiving attention because of their role in the degradation of recalcitrant biomass and their intriguing catalytic properties. The fundamentals of LPMO catalysis remain somewhat enigmatic as the LPMO reaction is affected by a multitude of LPMO- and co-substrate-mediated (side) reactions that result in a complex reaction network. We have performed kinetic studies with two LPMOs that are active on soluble substrates, *Nc*AA9C and *Ls*AA9A, using various reductants typically employed for LPMO activation. Studies with *Nc*AA9C under “monooxygenase” conditions showed that the impact of the reductant on catalytic activity is correlated with the hydrogen peroxide-generating ability of the LPMO-reductant combination, supporting the idea that a peroxygenase reaction is taking place. Indeed, the apparent monooxygenase reaction could be inhibited by a competing H_2_O_2_-consuming enzyme. Interestingly, these fungal AA9-type LPMOs were found to have higher oxidase activity than bacterial AA10-type LPMOs. Kinetic analysis of the peroxygenase activity of *Nc*AA9C on cellopentaose revealed a fast stoichiometric conversion of high amounts of H_2_O_2_ to oxidized carbohydrate products. A *k*_cat_ value of 124 ± 27 s^–1^ at 4 °C is 20 times higher than a previously described *k*_cat_ for peroxygenase activity on an insoluble substrate (at 25 °C) and some 4 orders of magnitude higher than typical “monooxygenase” rates. Similar studies with *Ls*AA9A revealed differences between the two enzymes but confirmed fast and specific peroxygenase activity. These results show that the catalytic site arrangement of LPMOs provides a unique scaffold for highly efficient copper redox catalysis.

## Introduction

Enzymes currently known as lytic polysaccharide monooxygenases (LPMOs) catalyze the oxidative scission of glycosidic bonds and by doing so they boost the activity of classical polysaccharide-degrading hydrolytic enzymes such as chitinases and cellulases.^1−10^ LPMO catalytic sites contain a single copper-ion cofactor^11,12^ that upon reduction reacts with either O_2_ or H_2_O_2_ to generate oxygen species that is capable of abstracting a hydrogen atom from the C1 or the C4 carbon atom in glycosidic bonds.^9,13−16^

**Figure 1:**
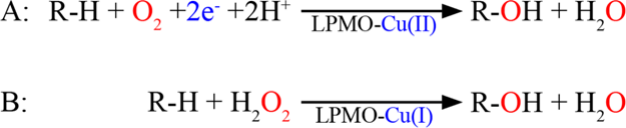
Reaction schemes for monooxygenase (A) and peroxygenase (B) reaction. The substrate is indicated by R. Hydroxylation of one of the carbons destabilizes the glycosidic bond, which, once oxidized, undergoes an elimination reaction leading to bond breakage.^12^ Note the potential difference in reductant consumption between the two reaction schemes. In the peroxygenase scheme, a once reduced LPMO can carry out multiple reactions,^20,36,37^ meaning that reductant consumption will be low if H_2_O_2_ is provided externally. If, however, H_2_O_2_ is generated *in situ* through the reduction of O_2_, also the peroxygenase reaction will require two electrons per cycle (O_2_ + 2e^–^ + 2H^+^ → H_2_O_2_).

Initially, LPMOs were thought to be monooxygenases^3^ (Figure 1A), but recent studies have shown that LPMOs can also act as peroxygenases^15^ (Figure 1B) and that this reaction is faster than the monooxygenase reaction.^15−20^ The peroxygenase reaction tends to lead to more enzyme damage compared to the monooxygenase reaction and may also lead to reduced catalytic specificity.^21^ Relatively rapid enzyme inactivation under peroxygenase conditions may be taken to indicate that the peroxygenase reaction is not a true LPMO reaction^21^ but could also have other explanations, such as sub-saturating substrate concentrations that leave the enzyme prone to damaging off pathway reactions with H_2_O_2_.^16,20,22^ Importantly, under the conditions typically used in LPMO “monooxygenase” reactions, H_2_O_2_ will be generated *in situ* and there are indications that the observed reaction rates in such reactions, typically in the range of a few per minute,^17^ reflect the rate of *in situ* generation of H_2_O_2_, rather than the rate of a true monooxygenase reaction.^23−25^*In situ* generation of H_2_O_2_ may result from LPMO-independent oxidation of the reductant by O_2_ and may also involve the LPMO because LPMOs have oxidase activity.^26−28^ These two routes toward H_2_O_2_ generation are intertwined in a manner that depends on both the reductant and the LPMO, whereas the impact of substrate binding on O_2_ activation^28−30^ adds an additional level of complexity. For example, Stepnov *et al.*(24) showed that the generation of H_2_O_2_ in standard reactions with an AA10 type (bacterial) LPMO (*i.e.*, LPMO + 1 mM reductant) was almost independent of the LPMO in reactions with gallic acid (GA), whereas the LPMO increased H_2_O_2_ production in reactions with ascorbic acid (AscA). It is not known whether the same would apply for the AA9 LPMOs that are abundant in biomass-degrading fungi.

Understanding LPMOs, which requires the robust assessment of LPMO kinetics, is complicated due to the many interconnected redox phenomena and catalytic pathways. In the presence of the substrate, LPMOs catalyze the oxidation of glycosidic bonds using O_2_ or H_2_O_2_ [mono-oxygenase or peroxygenase reaction; Figure 1].^9,14,15,30^ In the absence of a carbohydrate substrate, LPMOs catalyze the formation of H_2_O_2_ from molecular oxygen (oxidase reaction)^24,26^ and may also catalyze reactions of H_2_O_2_ with the reducing agent.^24,31^ The inhibitory effect of the substrate on H_2_O_2_ accumulation may reflect the inhibition of the oxidase reaction,^25^ as originally proposed by Kittl *et al.*,^26^ but may also reflect the consumption of the generated H_2_O_2_ in a productive LPMO reaction.^15^ Next to engaging in oxidase reactions, reduced LPMOs may act as an H_2_O_2_ scavenger in peroxidase-like reactions.^31^ Both these non-productive (per)oxidase reactions may lead to auto-catalytic enzyme inactivation.^15,20,22,32^

The substrate of most LPMOs is polymeric and insoluble, which complicates the determination of true substrate concentrations (*i.e.*, the concentration of productive binding sites) and generates kinetic complications related to potentially slow substrate association/dissociation. Slow substrate association is of particular importance because a reduced LPMO that is not bound to the substrate is prone to side reactions that may consume reactants and lead to enzyme damage, as outlined above.^15,32,33^ Interestingly, Hangasky *et al.*(21) showed that H_2_O_2_-consuming horse radish peroxidase (HRP), which has a soluble substrate, inhibited an LPMO acting on an insoluble substrate, while having only a minor inhibitory effect on an LPMO acting on a soluble substrate. This observation underpins the impact of the substrate on LPMO behavior, likely including impact on the activation of O_2_ and/or H_2_O_2_.^25,28−30^

In recent years, fungal AA9-type LPMOs active on soluble substrates have been discovered, including *Nc*AA9C from *Neurospora crassa*(34,35) and *Ls*AA9A from *Lentinus similis*.^29^ These enzymes, acting on a diffusible and easy to analyze substrate, provide a unique opportunity to kinetically assess the various LPMO reactions. Here, we present an in-depth kinetic analysis of *Nc*AA9C acting on cellopentaose, showing that this enzyme is a fast and specific peroxygenase, capable of reaching unprecedented high catalytic rates. Similar studies with *Ls*AA9A revealed differences between the two enzymes but confirmed that these AA9 type LPMOs are indeed competent peroxygenases. These results demonstrate the catalytic potential of the LPMO scaffold, which is higher than what could be anticipated when the first slow LPMO reactions were described.

## Materials and Methods

### Chemicals

All chemicals were, if not stated otherwise, purchased from Sigma-Aldrich, Thermo Fisher Scientific or VWR.

### Expression, Purification and Copper Saturation

Recombinant LPMO expression was done as previously described by Rieder *et al.*(38) In summary: the genes encoding *Ls*AA9A (UniProtKB: A0A0S2GKZ1) and *Nc*AA9C (UniProtKB: Q7SHI8) were codon optimized for *Pichia pastoris*, using the online tool provided by Thermo Fisher Scientific and cloned into the pBSYP_*GCW14*_Z plasmid, which facilitates constitutive expression and employs the native LPMO signal peptides for secretion. After *Smi*I linearization, the pBSYP_*GCW14*_Z-*LPMO* constructs were used for the transformation of killer plasmid-free *P. pastoris* BSYBG11 (Δ*AOX1*, Mut^S^) one-shot ready competent cells (Bisy GmbH, Hofstätten a.d. Raab, Austria) following the manual provided by the supplier.

For enzyme production, a single yeast colony was used to inoculate 500 mL of YPD [1% (w/v) Bacto yeast extract (BD Bioscience, San Jose, CA, USA), 2% (w/v) peptone from casein (tryptone) (Merck Millipore, Burlington, MA, USA) and 2% (w/v) glucose]. Incubation was performed over 60 h in a 2 l baffled shake flask at 120 rpm and 28 °C. The LPMO-containing supernatant was separated from the cells by centrifugation at 10,000*g* for 15 min at 4 °C and filtered using a 0.22 μm Steritop bottle-top filter (Merck Millipore, Burlington, MA, USA) prior to the concentration using a VivaFlow 200 tangential crossflow concentrator (molecular weight cutoff, MWCO, 10 kDa, Sartorius Stedim Biotech Gmbh, Germany) and Amicon Ultra centrifugal filters (MWCO 10 kDa, Merck Millipore, Burlington, MA, USA).

The LPMOs were purified using an Äkta purifier system (GE Healthcare Life Sciences, Uppsala, Sweden) equipped with a HiLoad 16/60 Superdex 75 size exclusion column (GE Healthcare Life Sciences, Uppsala, Sweden) that was equilibrated in 50 mM BisTris-HCl (pH 6.5), 150 mM NaCl. The single step size exclusion purification was performed at a flow rate of 1 mL/min. The protein content of the fractions was assessed by sodium dodecyl sulfate-polyacrylamide gel electrophoresis and fractions containing pure LPMO were pooled.

To ensure the copper saturation of the active site, the enzyme preparation was incubated for 1 h with a 3-fold molar excess of CuSO_4_ at 4 °C in 50 mM BisTris-HCl (pH 6.5) with 150 mM NaCl. Unbound copper was removed by three repetitions of buffer exchange to 50 mM BisTris-HCl (pH 6.5) using Amicon Ultra centrifugal filters (MWCO 10 kDa, Merck Millipore, Burlington, MA, USA). LPMO concentrations were determined using the Bradford protein assay with a bovine serum albumin as the standard. The copper saturated and purified proteins were stored in 50 mM BisTris-HCl (pH 6.5) at 4 °C until use.

*Af*AA11B, a chitin-active LPMO from *Aspergillus fumigatus* (UniProtKB: Q4WEH3), which will be described in detail elsewhere, was produced, purified, and copper saturated as described above for *Ls*AA9A and *Nc*AA9C.^38^ Copper-saturated chitin-active bacterial *Sm*AA10A (CBP21) was prepared as described previously.^39^

### LPMO Reactions with Soluble Substrates

All solutions used in activity assays were normal air-saturated solutions. LPMO reactions typically had a volume of 200 μL and were prepared in a 1.5 mL reaction tube with a conical bottom. Standard reactions contained 1 μL LPMO, 1 mM reductant, and 1 mM cellopentaose (95% purity; Megazyme, Wicklow, Ireland) in 50 mM BisTris-HCl (pH 6.5). Reactions supplemented with H_2_O_2_ contained typically 0.25 μM enzyme, 300 μM H_2_O_2_, 100 μM reductant, and 1 mM of the soluble substrate. Deviations from standard conditions were required for some experiments, as indicated in the appropriate figure legends. Stock solutions of 50 mM AscA (l-ascorbic acid, 99%, Simga-Aldrich), 10 mM GA (GA monohydrate ≥99%, Sigma-Aldrich), and 100 mM cysteine (l-cysteine ≥98%, Sigma-Aldrich) were prepared in ddH_2_O, aliquoted, and stored at −20 °C until use. 10 mM stock solutions of H_2_O_2_ (37% Merck) were prepared in pure water (TraceSELECT, Fluka) and stored at −20 °C until use. The H_2_O_2_ concentration was assessed by measuring the absorbance at 240 nm and using a molar extinction coefficient of 43.6 M^–1^ cm^–1^.

Because the order of mixing the various components of LPMO reactions matters, we started by mixing H_2_O, buffer stock solution, and the substrate followed by the LPMO. After incubation for 1 min at the desired temperature and rpm, the reaction was initiated by the addition of the reductant (time point zero). In case the reaction was supplemented with H_2_O_2_ or HRP (Sigma-Aldrich), these were added after the LPMO but before the pre-incubation step and before the addition of the reductant. Reactions were incubated either at 37 or 4 °C and at 750 rpm (ThermoMixer C, Eppendorf, Hamburg, Germany). For sampling, 25 μL of aliquots were withdrawn from the main reaction at regular time points. To quench the reaction and to achieve an appropriate dilution factor for subsequent HPAEC-PAD analysis of products (see below), 175 μL of 200 mM NaOH were added to each sample. For quantification with the Dionex ICS6000 system, the dilution factor was 1:40, due to a higher sensitivity of this system. Reactions with mannopentaose and xylopentaose (95% purity; Megazyme, Wicklow, Ireland) were set up and sampled in the same manner but were diluted 1:4 prior to HPAEC-PAD analysis.

The presented data points are the average values of at least three individual replicates and include the standard deviation, which is shown as a vertical line. Negative control reactions were performed by leaving out the reductant.

### Product Detection and Quantification

Reaction products were detected using high-performance anion exchange chromatography with pulsed amperometric detection (HPAEC-PAD). HPAEC was performed on a Dionex ICS5000 or ICS6000 system. The ICS5000 was equipped with a 3 × 250 mm CarboPac PA200 analytical column and a CarboPac PA200 guard column, and cello-oligomer containing samples were analyzed using a 26 min gradient, as described previously.^24^ For analysis with the ICS6000, we used a 1 × 250 mm CarboPac PA200 analytical column and a guard column of the same type. The flow rate during analysis was 63 μL*min^–1^ and the applied gradient was as follows: 1–14 min, from 1 to 100 mM potassium methanesulfonate (KMSA), concave; 14–17 min, washing step at 100 mM KMSA; 17–26 min, re-conditioning at 1 mM KMSA.

To assess the LPMO activity on cellopentaose, the generation of native cellobiose and cellotriose, which would proportionally increase with the C4-oxidized products, was quantified. Products from reactions with mannopentaose and xylopentaose were analyzed using a Dionex ICS5000 system in the configuration described above. For analysis of mannopentaose-containing samples, we used a 26 min gradient for the cellopentaose-containing samples. In case the reactions were set up with xylopentaose, we used a 39 min gradient as described elsewhere.^40^ Chromatograms were recorded and analyzed with Chromeleon 7, and plots were made using Microsoft Excel.

### H_2_O_2_ Production Assay

Hydrogen peroxide formation assays were performed as previously described by Kittl *et al.*(26) The reactions were performed in 96-well microplates with 100 μL of 50 mM BisTris/HCl buffer (pH 6.5) containing 1 μM LPMO, 100 μM Amplex Red (AR), 1% (v/v) DMSO, and 0.025 mg/mL HRP (final concentrations). After 5 min pre-incubation at 30 °C, the reactions were started by the addition of the 1 mM reductant (final concentration). The formation of resorufin was monitored over 30 min at 540 nm using a Multiskan FC microplate photometer (Thermo Fisher Scientific, Bremen Germany). Standard solutions for H_2_O_2_ quantification were supplemented with the reductant and if appropriate with 1 mM Glc_5_ to capture potential side reactions, as recently explained.^19,24^ The reductant and Glc_5_ were added prior the addition of HRP.

## Results and Discussion

### Reductant Influences the Apparent Monooxygenase Reaction

It is well known from earlier works that the reductant has a large impact on the efficiency of O_2_-driven LPMO reactions.^23,24,41,42^ In keeping with the monooxygenase paradigm, this dependency has been attributed to variation in the reductant’s ability to deliver electrons to the LPMO. As outlined above, considering the peroxygenase activity of LPMOs, it is conceivable that the observed variation also, or even primarily, reflects the reductant-dependent variation in the *in situ* synthesis of H_2_O_2_ during the reaction.^23,24^ Here, we addressed the impact of the reductant on *Nc*AA9C by studying the degradation of cellopentaose in the presence of AscA, cysteine, or GA. The reactions were performed using classical aerobic “monooxygenase” conditions with 1 μM enzyme, 1 mM Glc_5_, and 1 mM reductant.

**Figure 2:**
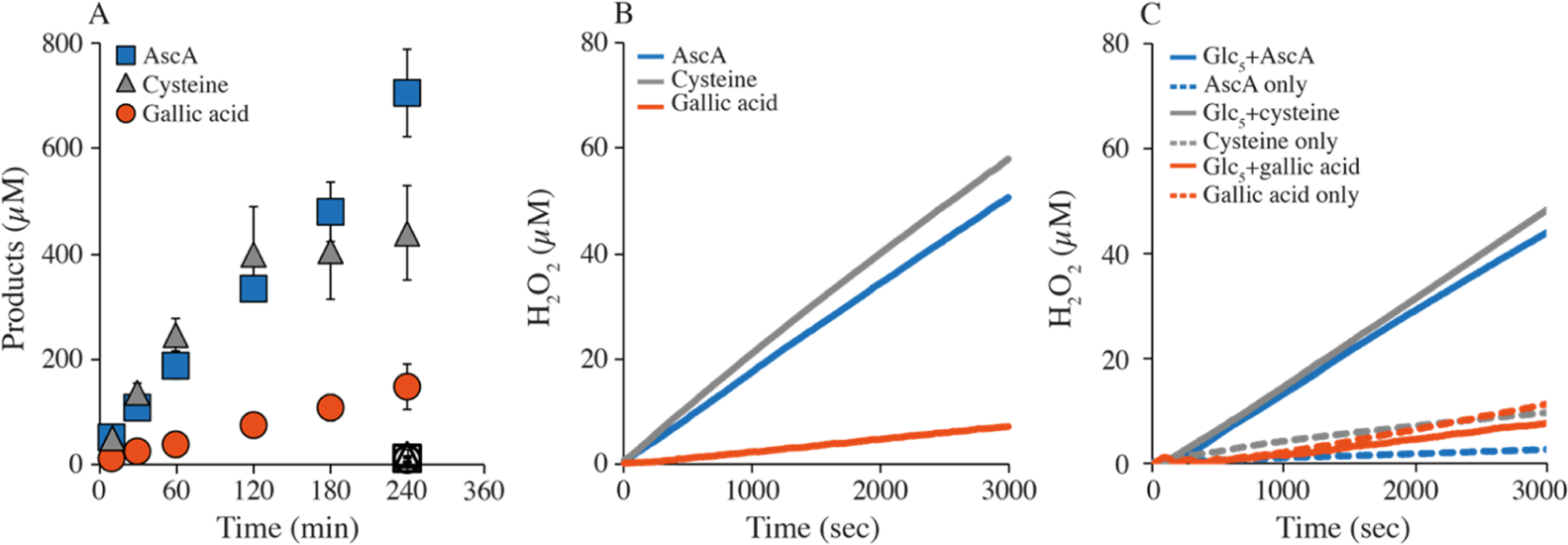
Progress curves showing the apparent monooxygenase (A) and oxidase (B) activity of *Nc*AA9C. Reactions were performed with 1 μM enzyme and 1 mM of either AscA (blue), cysteine (gray), or GA (orange) in the presence (A) or absence (B) of 1 mM Glc_5_. The empty symbols in A (at 240 min only) show the product levels (∼10 μM) found in the control reactions without a reductant. Panel (C) shows control reactions, that is, LPMO-independent H_2_O_2_ accumulation, in reactions with only reductant (dashed lines), or with reductant and 1 mM Glc_5_ (solid lines).

Figure 2A shows that stable reaction rates were obtained with AscA and GA, with apparent rate constants (*k*_obs_), derived from the linear part of the progress curves, of 0.05 ± 0.01 and 0.011 ± 0.02 s^–1^, respectively (Table 1). It is worth noting that the reaction with 1 mM AscA gave a linear progress curve up to at least the 800 μM oxidized product, which shows that the reaction was not O_2_ limited. The reaction with cysteine showed the highest initial rate (*k*_obs_ = 0.06 ± 0.01 s^–1^), but in this case product formation halted after approximately half of the substrate had been degraded. This is not surprising because, while AscA and GA can donate two electrons per molecule, cysteine can donate only one, meaning that two molecules of cysteine are needed per LPMO reaction and that 1 mM of cysteine can only fuel cleavage of 0.5 mM (*i.e.*, half) of the substrate.

**Table 1:** Apparent Rate Constants (s^–1^) for Reactions Catalyzed by *Nc*AA9C, with Three Different Reductants^a^

	mono-oxygenase (Figure 2A; 1 mM reductant, 1 mM Glc_5_, O_2_)	oxidase (Figure 2B; 1 mM reductant, O_2_, no substrate)	O_2_ reduction, reductant only, with substrate (Figure 2C; 1 mM reductant, 1 mM Glc_5_, O_2_, no LPMO)	O_2_ reduction, reductant only (Figure 2C; 1 mM reductant, O_2_, no LPMO)	peroxygenase (Figure 4A; 0.1 mM reductant, 1 mM Glc_5_, 300 μM H_2_O_2_, O2)
AscA	0.05 ± 0.01	0.017 ± 0.001	0.016 ± 0.000	0.0004 ± 0.0001	∼70^b^
GA	0.011 ± 0.002	0.002 ± 0.001	0.004 ± 0.000	0.0040 ± 0.0009	∼25^b^
cysteine	0.06 ± 0.01	0.019 ± 0.000	0.017 ± 0.000	0.0026 ± 0.0002	∼6

a The values presented are derived from the progress curves shown in Figures 2 and 4 and are either estimates based on the first time point (peroxygenase reaction) or represent the average of three individual replicates (mono-oxygenase and oxidase reaction).

b The shape of the progress curve in Figure 4A shows that this rate is underestimated.

To gain insights into the oxidase activity of *Nc*AA9C and a possible connection between this activity and the enzyme’s apparent mono-oxygenase activity, we measured H_2_O_2_ production in the absence of the substrate using the AR/HRP assay, as described previously.^24,26^ Of note, while this assay is very useful, it suffers from multiple complications (discussed in, *e.g.*, refs (19) and (24)) that prevent extrapolation of apparent H_2_O_2_ production levels in a reaction without the substrate (Figure 2B) with expected H_2_O_2_ production levels in a reaction with the substrate (Figure 2A). First, the reductant suppresses the signal of the HRP assay and this will vary between reductants. Although the reductant is included in the standard curve for H_2_O_2_, this effect cannot be fully compensated for.^19,24^ Second, H_2_O_2_ may react with the reductant (meaning that H_2_O_2_ levels will be underestimated) and this reaction may be promoted by HRP to an extent that differs between the reductants; this situation will be entirely different in a reaction with the substrate, where the productive LPMO reaction will outcompete slower background reactions with the reductant. Finally, as alluded to above, the presence of the substrate inhibits the oxidase activity of the LPMO.^25,26,34^

Figure 2B and the derived reaction rates (Table 1) show that apparent H_2_O_2_-production rates vary between the reductants, showing trends that align well with apparent mono-oxygenase reaction rates (Figure 2A; Table 1). The apparent mono-oxygenase activity is about 5 times higher with AscA and cysteine than with GA. The variation in the apparent oxidase rates shows a similar trend, but in this case, the rate difference between AscA/cysteine and GA is about 10-fold. For all reductants, the apparent mono-oxygenase activity is 3 to 5 times higher than the apparent oxidase activity, which could indicate that we indeed are observing mono-oxygenase activity in a reaction that is not limited by the generation of H_2_O_2_. However, this phenomenon could also be due to the underestimation of H_2_O_2_ production for reasons described above, and addressed further below, or be caused by an additional source of H_2_O_2_ in reactions with the substrate, Glc_5_, as discussed below.

Intrigued by the difference between the apparent mono-oxygenase and oxidase activities, we investigated a possible effect of 1 mM Glc_5_ on H_2_O_2_ production in reactions with standard amounts of all three reductants. The obtained results show that, for reactions with AscA and cysteine, incubation of Glc_5_ with the reductant led to strongly increased H_2_O_2_ production, relative to reactions with only reductant (Figure 2C). The apparent H_2_O_2_ production rates in these reactions were not unlike the rates obtained in reactions with the reductant and LPMO (Figure 2B) and are thus quite significant (Table 1). This unexpected effect of Glc_5_ could be due to the presence of transition metals, likely copper, which would enhance H_2_O_2_ production through the oxidation of AscA^24,43^ and cysteine,^44^ but not necessarily of GA^24^ because GA is more likely to form complexes with Cu(II) rather than reducing it.^45^ This additional source of H_2_O_2_ helps to close the gap observed between the rates of the apparent mono-oxygenase and oxidase activities.

Of note, the results depicted in Figure 2 show that the combination of *Nc*AA9C and GA is not suitable for the assessment of LPMO oxidase activity by the AR/HRP assay as the apparent rate of H_2_O_2_ production in reactions with GA alone (Figure 2C, Table 1) is higher that the apparent oxidase activity in reactions with GA and the LPMO (Figure 2B; Table 1). In this case, the assay is flawed due to the ability of *Nc*AA9C to engage in a H_2_O_2_-consuming side reaction with GA, as described by Breslmayr *et al.*(31) Of note, in a LPMO reaction mixture containing Glc_5_, side reactions with GA will be outcompeted by the peroxygenase reaction with Glc_5_, which is faster, as shown below.

A recent study on a cellulose-active AA10-type LPMO with AscA and GA as reductants showed that the LPMO had little effect on H_2_O_2_ production, which was dominated by the LPMO-independent oxidation of the reductant.^24^Table 1 shows that the situation for *Nc*AA9C is different. In this case, the LPMO may contribute considerably to apparent H_2_O_2_ production in reactions with cysteine and AscA (compare “oxidase” with “O_2_ reduction, reductant only”). In the case of AscA, the LPMO speeds up the H_2_O_2_ production rate by some 40-fold, whereas the increase is some 7-fold for cysteine. Similar comparisons for GA could not be made due to the technical issues discussed above.

**Figure 3:**
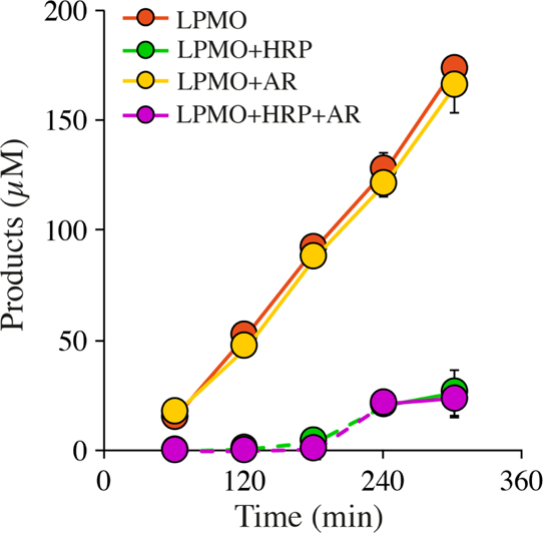
HRP inhibition for reactions with *Nc*AA9C and GA. Progress curves showing product formation by 1 μM LPMO in the presence of 1 mM GA and 1 mM Glc_5_ with no supplementation (orange) or supplemented with 100 μM AR (yellow) or 2 μM HRP (green) or both (purple). Note that the HRP reaction does not depend on AR because GA is a substrate for HRP (see text). Dashed lines connect points with values that were close to the limit of detection.

If it is the *in situ* generation of H_2_O_2_ that is limiting the apparent mono-oxygenase reaction in the presence of GA, it should be possible to inhibit the LPMO reaction with another H_2_O_2_-consuming enzyme. Indeed, both Bissaro *et al.*(15) and Hangasky *et al.*(21) have shown that LPMO reactions with insoluble substrates under “mono-oxygenase conditions” are inhibited when adding HRP and its substrate, AR. While Hangasky *et al.* did not observe similarly strong inhibition in a reaction with a soluble substrate, Figure 3 shows that HRP strongly inhibits the GA-driven activity of *Nc*AA9C on Glc_5_. A similar degree of inhibition was observed in the reaction-containing HRP but lacking AR, indicating that HRP can oxidize GA, which is not surprising considering the literature data.^46^ Of note, it is highly unlikely that the LPMO inhibition in the presence of HRP is driven by reductant depletion rather than by competition for H_2_O_2_, given the high (1 mM) reductant concentration used in the experiment. Note that the observed side reaction between HRP and GA will also occur in the AR/HRP assay, contributing to the underestimation of the apparent H_2_O_2_ production rates derived from Figure 2.

### Peroxygenase Reaction Is Dependent on the Reductant

**Figure 4:**
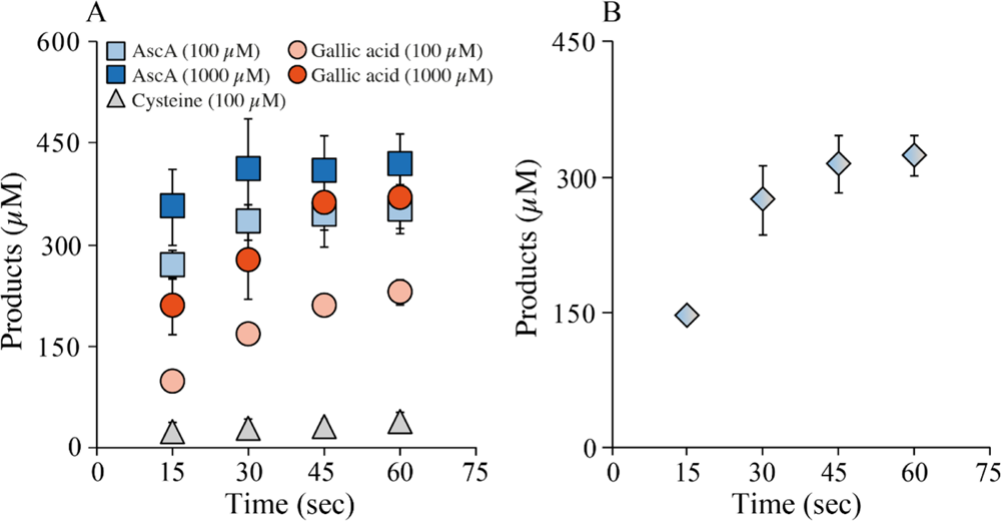
Peroxygenase reactions with *Nc*AA9C. (A) Time course experiments showing the impact of AscA (blue), cysteine (gray), and GA (orange) on the peroxygenase reaction catalyzed by *Nc*AA9C. Reaction mixtures containing 0.25 μM enzyme, 300 μM H_2_O_2_, 100 or 1000 μM reductant and 1 mM Glc_5_ were incubated at 37 °C and reactions were started by adding the reductant. No products were detected in control reactions without an added reductant. (B) Product formation in a reaction with 0.25 μM *Nc*AA9C, 300 μM H_2_O_2_, 1 mM Glc_5_, 0.1 mM AscA, and 0.1 mM cysteine. Note that this reaction was incubated at 4 °C, hence the slower rate compared to panel A.

To assess the influence of AscA, GA, and cysteine on the peroxygenase activity of *Nc*AA9C, we monitored the consumption of Glc_5_ in reactions that contained 300 μM H_2_O_2_ (Figure 4A). In the presence of the 100 μM reductant, we observed apparent rate constants of ∼70, ∼25, and ∼6 s^–1^ for AscA, GA, and cysteine, respectively, where the first and the second values are underestimated as a major part of H_2_O_2_ was consumed at the first time point. These rates are 100–2300 times higher than the apparent monooxygenase rates (Table 1). The progress curve for the reaction with AscA shows that the reaction is limited by the availability of H_2_O_2_ as product formation levels of at about 300 μM of the product, reflecting a 1:1 ratio with the added H_2_O_2_. It is worth noting that these reactions were monitored by measuring the generation of cellobiose and cellotriose, which means that uncertainties related to the instability of C4-oxidized products^40^ were avoided. It is also worth noting that reactions with a starting concentration of 300 μM H_2_O_2_ would lead to rapid LPMO inactivation in reactions with an insoluble substrate^15^ but that in the present case, with a rapidly diffusing soluble substrate, stoichiometric catalytic conversion of the H_2_O_2_ was achieved.

To investigate if the availability of the reductant is rate limiting, the experiments depicted in Figure 4A are redone with 1 mM (*i.e.*, 10-fold more) reductant concentrations. By doing so, the already high and most certainly underestimated rate for the reaction with AscA increased slightly, whereas the reaction with GA became approximately twice as fast. While this clearly shows that the reductant to some extent limits, the very high rates of these peroxygenase reactions (note the difference in time scale with the mono-oxygenase reactions of Figure 2), increasing the amount of the reductant had no effect on the (lower) rate of the reaction with cysteine (results not shown). The lower activity with cysteine was not due to H_2_O_2_ scavenging by the reductant, as an addition of 0.1 mM cysteine to a reaction with 0.1 mM AscA did not affect product formation (Figure 4B), which shows that all the added H_2_O_2_ was used by the LPMO. This result is in line with the literature data showing that, while cysteine can react with H_2_O_2_, the rate of this reaction is orders of magnitude lower^47^ than the rate of the peroxygenase reaction of *Nc*AA9C. Possibly, the reduction of copper by cysteine leads to the formation of a relatively stable cuprous thiolate complex^48^ that limits LPMO reactivity under “fast” peroxygenase conditions, whereas this inhibitory effect could remain unnoticed under much slower mono-oxygenase conditions. Of note, even with cysteine, a *k*_obs_ of ∼6 s^–1^ is still much higher than typical *k*_obs_ values for mono-oxygenase reactions.

These results show that the peroxygenase reaction of *Nc*AA9C is much faster than the apparent mono-oxygenase reaction (Table 1), which implies that minor variations in the levels of *in situ* H_2_O_2_ generation will have dramatic effects on the low rates of the latter reaction. Within the time scale of the peroxygenase reaction, the main contribution of the reductant is to keep the LPMO reduced (*i.e.*, catalytically competent) and our data reveal differences between the reductants in this respect. While the experiments with polymeric substrates have shown that once reduced LPMOs may catalyze 15–20 peroxygenase reactions before being re-oxidized,^20,36,37^ the re-oxidation frequency, and, thus, the reductant dependency may be higher in the case of a soluble substrate, which will bind less strongly and, upon binding, create less confinement of the copper site, thus increasing the chances for side reactions that involve copper reoxidation and the loss of electrons.

### Kinetics of the LPMO-Catalyzed Peroxygenase Reaction

**Figure 5:**
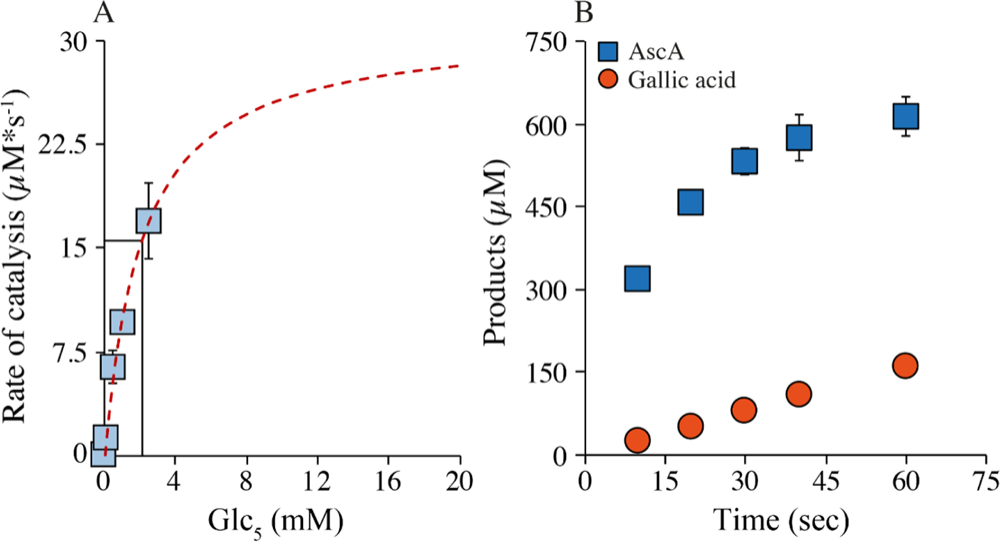
Kinetics of the *Nc*AA9C-catalyzed peroxygenase reaction with Glc_5_. (A) Michealis–Menten kinetics showing the dependency of the catalytic activity on the Glc_5_ concentration. The rates were derived from linear progress curves and the dashed line shows the fit to the Michalis–Menten equation. Reactions were set up with a 0.25 μM enzyme and 600 μM H_2_O_2_ at 4 °C and were started by adding 0.1 mM AscA (note that the *K*_m_ for H_2_O_2_ is expected to be below 10 μM^18^). (B) Progress curves for the peroxygenase reaction at 4 °C. The data points show product formation in a reaction with 0.25 μM *Nc*AA9C, 600 μM H_2_O_2_, 1 mM Glc_5_, and either 1 mM AscA (blue) or GA (orange) that was incubated at 4 °C.

To gain more insights into the peroxygenase reaction, we performed Michaelis–Menten kinetics (Figure 5A). The underlying linear progress curves covered Glc_5_ concentrations ranging from 75 to 2500 μM and reactions were run at 4 °C to obtain manageable product formation rates. This setup resulted in a hyperbolic curve, yielding a *K*_m_ (for Glc_5_) of 2.1 ± 0.3 mM, a *k*_cat_ of 124 ± 27 s^–1^, and a *k*_cat_/*K*_m_ of 5.9*10^4^ M^–1^ s^–1^ (Figure 5A). This *k*_cat_ value, determined at 4 °C, is 2.5 × 10^3^-fold higher than the *k*_obs_ value for the apparent mono-oxygenase reaction with AscA described above (37 °C), 1.1 × 10^3^-fold higher than the *k*_cat_ value reported for *Ls*AA9A acting on an analogue of Glc_4_ in a mono-oxygenase setup with AscA (37 °C),^29^ and 19-fold higher than the *k*_cat_ reported for a peroxygenase action on chitin nanowhiskers by a bacterial AA10-type LPMO at 25 °C.^18^ The *K*_m_ measured for *Nc*AA9C of ∼2 mM is comparable to a *K*_d_ of 0.81 ± 0.08 mM that Borisova *et al.*(35) measured for the same enzyme binding to Glc_6_ under non-turnover conditions.

To further substantiate the strikingly high catalytic rate of *Nc*AA9C, we then conducted additional initial rate measurements to obtain *k*_obs_ values that would be more reliable than those obtained from the non-linear progress curves shown in Figure 4A. To do so, we decreased the reaction temperature to 4 °C and increased the H_2_O_2_ concentration to 600 μM to ensure that the oxygen-donating substrate would not become limiting within seconds. The resulting progress curve for the reaction with AscA (Figure 5B) showed the formation of 600 μM products within 30 s showing that the reaction was limited by the availability of H_2_O_2_. Based on the first 20 s of the experiment (*R*^2^ = 0.95), we calculated a *k*_obs_ of 90.8 ± 3.6 s^–1^. As expected, based on Figure 4A, the reaction with GA was slower. This reaction showed a linear increase in the product level and gave a *k*_obs_ of 10.7 ± 0.3 s^–1^ (Figure 5B). Of note, these rates were obtained using sub-saturating substrate conditions as the used Glc_5_ concentration was just about 50% of the measured *K*_m_. Still the obtained *k*_obs_ of ∼90 and ∼11 s^–1^ for *Nc*AA9C in combination with AscA and GA, respectively, represent the two highest rates ever measured for the LPMO-catalyzed oxidation of a carbohydrate substrate.

### AA9 LPMOs Acting on Soluble Substrates Have Different Properties

One of the other AA9 LPMOs known to act on soluble substrates is *Ls*AA9A.^29^ A previous kinetic characterization of this enzyme using a Förster-resonance energy-transfer (FRET) substrate analogue of Glc_4_ as a substrate and mono-oxygenase conditions (5 mM AscA, no added H_2_O_2_) yielded a *k*_cat_ = 0.11 ± 0.01 s^–1^, that is, a typical value for LPMOs acting in the “mono-oxygenase mode”, and in the same range as apparent oxidase and mono-oxygenase rates reported here for reactions with AscA (Table 1). The obtained *K*_m_ value of 43 ± 9 μM is remarkably low, compared to, for example, the *K*_m_ for Glc_5_ cleavage by *Nc*AA9C reported above and suggests high substrate affinity, which could perhaps be due in part to the presence of aromatic groups that appear at the reducing and non-reducing ends of the FRET substrate analogue.

**Figure 6:**
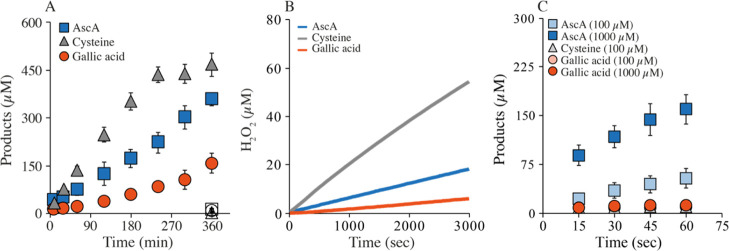
Mono-oxygenase, peroxygenase, and oxidase activity for *Ls*AA9A. Mono-oxygenase (A) and oxidase (B) reactions were performed with 1 μM LPMO and either 1 mM AscA (blue), cysteine (gray), or GA (orange) in presence (A) and absence (B) of 1 mM Glc_5_. The empty symbols in A (at 360 min only) show the product levels found in the control reactions without a reductant. For the peroxygenase reactions shown in panel (C), we lowered the enzyme concentration to 0.25 μM and added 300 μM H_2_O_2_ with the same reductants as used for the mono-oxygenase reaction at concentrations of either 100 or 1000 μM at 37 °C. In panel C, the points for the reaction with 100 μM GA and cysteine, respectively, are hidden by the points for the reaction with 1000 μM GA; the reaction with 1000 μM cysteine yielded the same curve as the reaction with 100 μM and is not shown, for clarity.

Our studies confirmed high substrate affinity, albeit not necessarily specific, as we observed increasing substrate inhibition (*i.e.*, an increasing reduction of LPMO activity) at Glc_5_ concentrations above 0.1 mM (results not shown). Due to this substrate inhibition, a quantitative comparison of the catalytic properties of the two LPMOs is not straightforward. Assays identical to those described above for *Nc*AA9C showed apparent mono-oxygenase and oxidase rates in the same order of magnitude and confirmed the considerable impact of the reductant of LPMO activity (Figure 6; Table 2). The most notable difference is that H_2_O_2_ production by *L*sAA9A in the presence of AscA is less efficient compared to *Nc*AA9C (Figures 2B and 6B). Accordingly, the AscA-driven apparent mono-oxygenase reaction is slower, making cysteine the clearly most efficient reductant for this LPMO in a “mono-oxygenase” setup (Figure 6A).

**Table 2:** Apparent Rate Constants (s^–1^) for the Oxidation of 1 mM Glc_5_ by *Ls*AA9A under Various Conditions^a^

	mono-oxygenase (Figure 6A; 1 mM reductant, 1 mM Glc_5_, O_2_)	oxidase (Figure 6B; 1 mM reductant, O_2_, no substrate)	O_2_ reduction, reductant only (Figure 2C; 1 mMreductant, O_2_, no LPMO)	peroxygenase (Figure 6C; 0.1 mM reductant, 1 mM Glc_5_, 300 μM H_2_O_2_, O_2_)	peroxygenase (Figure 6C; 1 mM reductant, 1 mM Glc_5_, 300 μM H_2_O_2_, O_2_)
AscA	0.014 ± 0.002	0.006 ± 0.000 (35%)	0.0004 ± 0.0001	5.8 ± 2.3	23.4 ± 4.2
GA	0.006 ± 0.001	0.002 ± 0.000 (100%)	0.0040 ± 0.0009	0.1 ± 0.0	0.4 ± 0.1
cysteine	0.029 ± 0.001	0.018 ± 0.000 (95%)	0.0026 ± 0.0002	0.3 ± 0.1	0.2 ± 0.1

a The values presented are estimates derived from the progress curves shown in Figure 6. The oxidase values are also expressed as a percentage of the oxidase value observed for *Nc*AA9C (Table 1). Other quantitative comparisons between the two LPMOs are not straightforward due to the occurrence of substrate inhibition in the reactions with *Ls*AA9A.

The peroxygenase reactions were slower than for *Nc*AA9C, possibly due to substrate inhibition (Figure 6C). Still, the apparent rates recorded for reactions with two concentrations of AscA (Table 2) are 35–141 times higher than the previously determined *k*_cat_ for an apparent mono-oxygenase reaction^29^ and 280–1100 times higher than the apparent mono-oxygenase reaction rates determined here. For this LPMO, peroxygenase reactions with both cysteine and GA were relatively slow and not or hardly dependent on the reductant concentration. Still, these rates were some 10 and 100 times higher than the determined apparent mono-oxygenase rates (Table 2).

**Figure 7:**
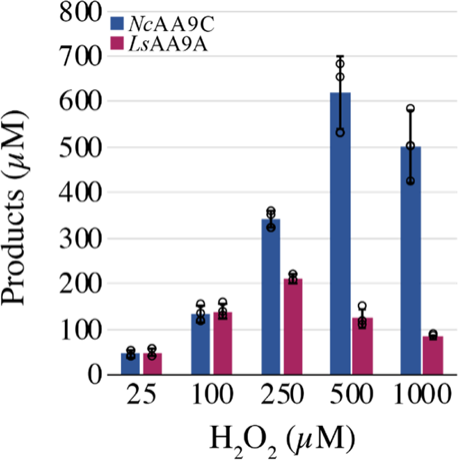
Sensitivity of *Nc*AA9C and *Ls*AA9A for oxidative damage. The graph shows product levels obtained after a 2 min reaction containing 1 mM AscA and various amounts of H_2_O_2_. Reaction mixtures containing 1 μM *Ls*AA9A (purple) or 1 μM *Nc*AA9C (blue), 1 mM Glc_5_, and varying H_2_O_2_ concentrations (25–1000 μM) were pre-incubated for 1 min, after which the reaction was started by adding the reductant. In reactions not showing signs of enzyme inactivation, product levels were slightly higher than the amount of added H_2_O_2_ due to the combination of AscA-mediated H_2_O_2_ generation and a small systematic error in the concentration of the H_2_O_2_ stock solution.

It is interesting to note that the efficient peroxygenase reaction catalyzed by *Ls*AA9A in the presence of AscA was much more dependent on the reductant concentration (Figure 6C; Table 2) compared to *Nc*AA9C (Figure 2). This reflects that, compared to *Nc*AA9C, *Ls*AA9A is more prone to oxidation and a subsequent need for re-reduction. Substrate binding and the resulting confinement of the reduced catalytic copper form a major determinant of the degree of non-productive LPMO oxidation. The data could thus indicate that *Ls*AA9A binds the substrate less for firmy or less precisely, where the first option is in conflict with the previously reported low *K*_m_ value. A more oxidation-prone copper site in the enzyme–substrate complex would also translate into decreased enzyme stability at higher H_2_O_2_ concentrations, as non-productive reactions between the reduced enzyme and H_2_O_2_ may lead to oxidative damage.^15^ Indeed, Figure 7 shows that *Ls*AA9A is more sensitive to H_2_O_2_-induced damage than *Nc*AA9C. While product formation by *Nc*AA9C first started decreasing at 1000 μM, the highest tested H_2_O_2_ concentration, *Ls*AA9A, showed signs of enzyme inactivation already at 250 μM (Figure 7).

As a cautionary note, we cannot exclude that the non-natural glycosylation of the *Pichia*-produced LPMOs may affect their properties. Considering the predicted location of glycosylation sites and the crystal structure of the *Pichia*-produced protein,^35^ such an effect of glycosylation can be excluded for *Nc*AA9C. Based on the predicted glycosylation sites, glycosylation effects on the interaction between *Ls*AA9A and Glc_5_ seem unlikely but cannot be excluded. Assuming that glycosylation effects do not play a role, the comparison of the results obtained for *Nc*AA9C and *Ls*AA9A show two important things. First, the data reveal functional differences between these two C4-oxidizing cellulose-active LPMOs, which are reductant dependent. Because soluble cello-oligomers can easily be degraded by hydrolytic enzymes, it is not likely that nature has evolved LPMOs for the purpose of cleaving these compounds (as also suggested by the high *K*_m_ value for *Nc*AA9C). Therefore, we hypothesize that the functional differences between *Nc*AA9C and *Ls*AA9A should be considered as a proxy for hitherto undescribed differences in substrate preferences that relate to the structural and compositional complexity of the true biomass. Second, while our studies show quite different peroxygenase reaction rates and reductant dependencies for *Nc*AA9C and *Ls*AA9A and they suggest that Glc_5_ is not an optimal substrate for *Ls*AA9A, all the observed peroxygenase rates are much higher than any reported apparent rate for apparent mono-oxygenase reactions.

### LPMO-Catalyzed Peroxygenase Reaction Is Specific

**Figure 8:**
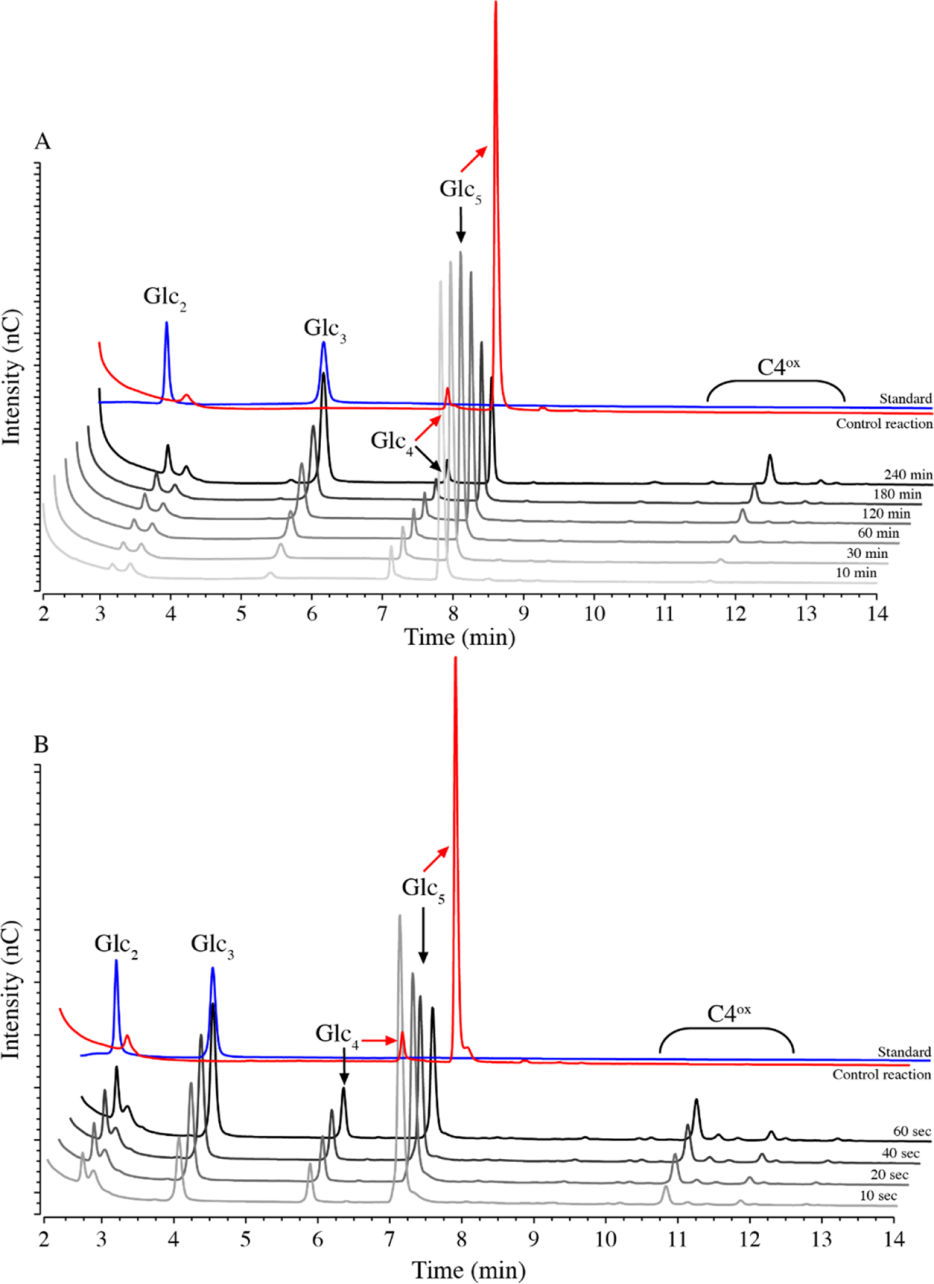
HPAEC-PAD chromatograms showing product formation in reactions with *Nc*AA9C and Glc_5_, using mono-oxygenase (A) or peroxygenase (B) setup. Chromatograms for the mono-oxygenase reaction (1 μM LPMO, 1 mM AscA, and 1 mM Glc_5_ at 37 °C) and peroxygenase reaction (0.25 μM LPMO, 600 μM H_2_O_2_, 1 mM AscA, and 1 mM Glc_5_ at 4 °C) are shown as lines in gradations of gray and black. The chromatograms correspond to the time course experiments shown in Figures 2A and 5B, respectively. The red lines show the chromatograms of the appropriate control reaction without a reductant, after incubation for 240 min (A) or 60 s (B). The blue chromatograms show the Glc_2_ + Glc_3_ standard.

Previously, it has been claimed that the addition of H_2_O_2_ to LPMO reactions results in a loss in specificity^21^ and some argue that this shows that H_2_O_2_ is not a bonafide co-substrate for LPMOs and that, thus, LPMOs are not bonafide peroxygenases. In the present study, we used high H_2_O_2_ amounts that were stoichiometrically used to convert cellopentaose to cellobiose and cellotriose. This shows that there is little, if any, random oxidation of the substrate and that the reaction is specific (Figure 8).

To further assess specificity, we set up aerobic reactions with 1 μM *Ls*AA9A or 1 μM *Nc*AA9C with either 1 mM xylopentaose (Xyl_5_) or 1 mM mannopentaose (Man_5_) as a substrate (Figure S1; Figure S2). The conditions used were as follows: (i) 1 mM AscA (“mono-oxygenase” conditions), (ii) 20 μM H_2_O_2_ and 20 μM AscA, or (iii) 300 μM H_2_O_2_ and 100 μM AscA. Note that the latter reaction conditions would lead to very fast (within < 1 min) conversion of Glc_5_ by *Nc*AA9C (Figure 4A). Additionally, we tested well-characterized chitin-active *Sm*AA10A^3^ and a recently described chitin-active AA11, called *Af*AA11B^38^ for their ability to oxidize 1 mM Glc_5_ using the same reaction conditions (Figure S3).

None of these reactions yielded a detectable turnover of the substrate, except the positive control reactions with *Nc*AA9C or *Ls*AA9A and Glc_5_ (Figure S3). We were not able to detect any degradation products by MALDI-TOF MS, whereas the HPAEC-PAD chromatograms only showed a few minimal signals that could indicate a low level of an oxidative cleavage of xylopentaose, which, for *Ls*AA9A, would be in accordance with a previously observed weak xylan-degrading ability.^49^ Crystallographic studies have shown that xylopentaose binds atypically to *Ls*AA9A, leaving a not properly confined copper site prone to engaging in potentially enzyme-inactivating side reactions.^10,49^ One would thus expect rapid enzyme inactivation in reactions with large amounts of H_2_O_2_, which could explain why, if at all present, only trace amounts of LPMO products were observed.

The main take home message of these experiments is that the addition of H_2_O_2_ at low or high concentration, in combination with different concentrations of AscA, does not result in a loss of substrate specificity. The chromatograms and mass spectra for the peroxygenase reactions did not show any conspicuous features compared to the negative controls or the chromatograms for the apparent mono-oxygenase reactions.

## Concluding Remarks

The experiments described above show two important aspects of LPMO enzymology. First, they illustrate that it is complicated to properly assess LPMO catalysis experimentally, due to the plethora of interconnected (side) reactions. Many of these complications emerged in our experiments and by studying multiple reductants, each with its own peculiarities, we were able to overcome most of these complications and generate insights into LPMO catalysis. Second, we show that LPMOs, when acting on rapidly diffusing soluble substrates and provided with H_2_O_2_, indeed are very efficient peroxygenases. We observed a stoichiometric conversion of high starting amounts of H_2_O_2_ that would lead to rapid LPMO inactivation in reactions with an insoluble substrate. Our data for reactions with soluble substrates show that the peroxygenase reaction is stable and specific.

We observed a correlation between the H_2_O_2_-producing potential of an LPMO-reductant combination and the observed apparent monooxygenase activity, which supports the idea that, under these conditions, the rate of the apparent monooxygenase reaction may reflect the rate of an H_2_O_2_-limited peroxygenase reaction, as originally suggested by Bissaro *et al.*(15) This is supported by the strong inhibitory effect of HRP on the LPMO reaction. We cannot exclude that a monooxygenase reaction also occurs, and it is well known that reduced LPMOs react with O_2_.^13,26^ It is also known that this reaction may be influenced by substrate binding.^29,30^ The rates of the two reactions vary a lot for both soluble and insoluble substrates (refs (16)–^19^; this study) and here, we show that peroxygenase reactions with a soluble substrate may reach rates in an order of 100 s^–1^.

Notably, our data indicate that the oxidase activity of the AA9 type LPMOs studied here is higher that the oxidase activity of a previously studied AA10 type LPMO.^24^ This could imply that, compared to AA10 LPMOs, the AA9 LPMOs are more active under monooxygenase conditions than AA10 LPMOs because they generate more H_2_O_2_. However, the extrapolation of oxidase activities measured in the absence of the substrate to oxidase activities under turnover conditions is not straightforward because of the impact of substrate binding on oxidase activity.^25^ Further studies are warranted to study whether the observed difference in oxidase activity is general and to identify its structural determinants. It is also worth noting that in systems where the LPMO peroxygenase reaction is driven by the oxidase activity of the LPMO itself, the nature of the reductant will have a decisive impact on LPMO efficiency.

Our study revealed differences between *Nc*AA9C and *Ls*AA9A, which suggests that these enzymes have different substrate specificities and biological roles. It is important to realize that laboratory experiments with substrates such as Glc_5_ or pure cellulose only give limited insights into the true role of an LPMO during fungal biomass conversion.

The most important and novel findings of the present study is that the unique LPMO scaffold enables highly efficient copper-catalyzed peroxygenase reactions with a soluble substrate. This high efficiency may in part be due to the copper site being exposed and rather rigid, with an open coordination position for co-substrate binding.^50^ Thus, as originally pointed out by Kjaergaard *et al.*,^13^ catalysis requires little reorganization energy, which may contribute to efficiency. It is encouraging that high specificity and high catalytic rates were achieved with what seems to be a low affinity substrate. It may be possible to engineer similar or better affinities for other, perhaps non-carbohydrate, substrates, which eventually could endow these powerful enzymes with the ability to catalyze efficient peroxygenation of such substrates. Furthermore, the unique peroxygenase chemistry of these mono-copper enzymes may open new avenues for the future design of enzyme-inspired synthetic copper catalysts.

## Supporting Information Available

HPLC product profiles for reactions of *Nc*AA9C or *Ls*AA9A with xylopentaose; HPLC product profiles for reactions of *Nc*AA9C or *Ls*AA9A with mannopentaose; and HPLC product profiles for reactions of *Af*AA11B or *Sm*AA10A with cellopentaose (PDF)

## Accession Codes

*Nc*AA9C, a family AA9 LPMO from *Neurospora crassa*, UniProt Q7SHI8; *Ls*AA9A, a family (AA) LPMO from *Lentinus similis*, UniProt A0A0S2GKZ1; *Af*AA11B, a family AA11 LPMO from *Aspergillus fumigatus*, UniProt Q4WEH3; *Sm*AA10A, a family AA10 LPMO from *Serratia marcescens*, UniProt O83009.
