# Supplementary material for: Fast and Specific Peroxygenase Reactions Catalyzed by Fungal Mono-Copper Enzymes
Source: Biochemistry. 2021 Nov 5;60(47):3633–43. doi: 10.1021/acs.biochem.1c00407 (PMC8638258; doi:10.1021/acs.biochem.1c00407)
Supplement: Supplementary file 1 — bi1c00407_si_001.pdf [file bi1c00407_si_001.pdf]

Fast and specific peroxygenase reactions catalyzed by fungal mono-copper enzymes

Lukas Rieder, Anton A. Stepnov, Morten Sørlie, Vincent G.H. Eijsink*

Faculty of Chemistry, Biotechnology, and Food Sciences, Norwegian University of Life Sciences (NMBU), P.O. Box 5003, NO-1432, Ås, Norway

* For correspondence; E-mail: vincent.eijsink@nmbu.no

Supporting information

Table of contents

Figure S1: HPLC product profiles for reactions of *Nc*AA9C or *Ls*AA9A with xylopentaose

Figure S2: HPLC product profiles for reactions of *Nc*AA9C or *Ls*AA9A with mannopentaose

Figure S3: HPLC product profiles for reactions of *Af*AA11B or *Sm*AA10A with cellopentaose

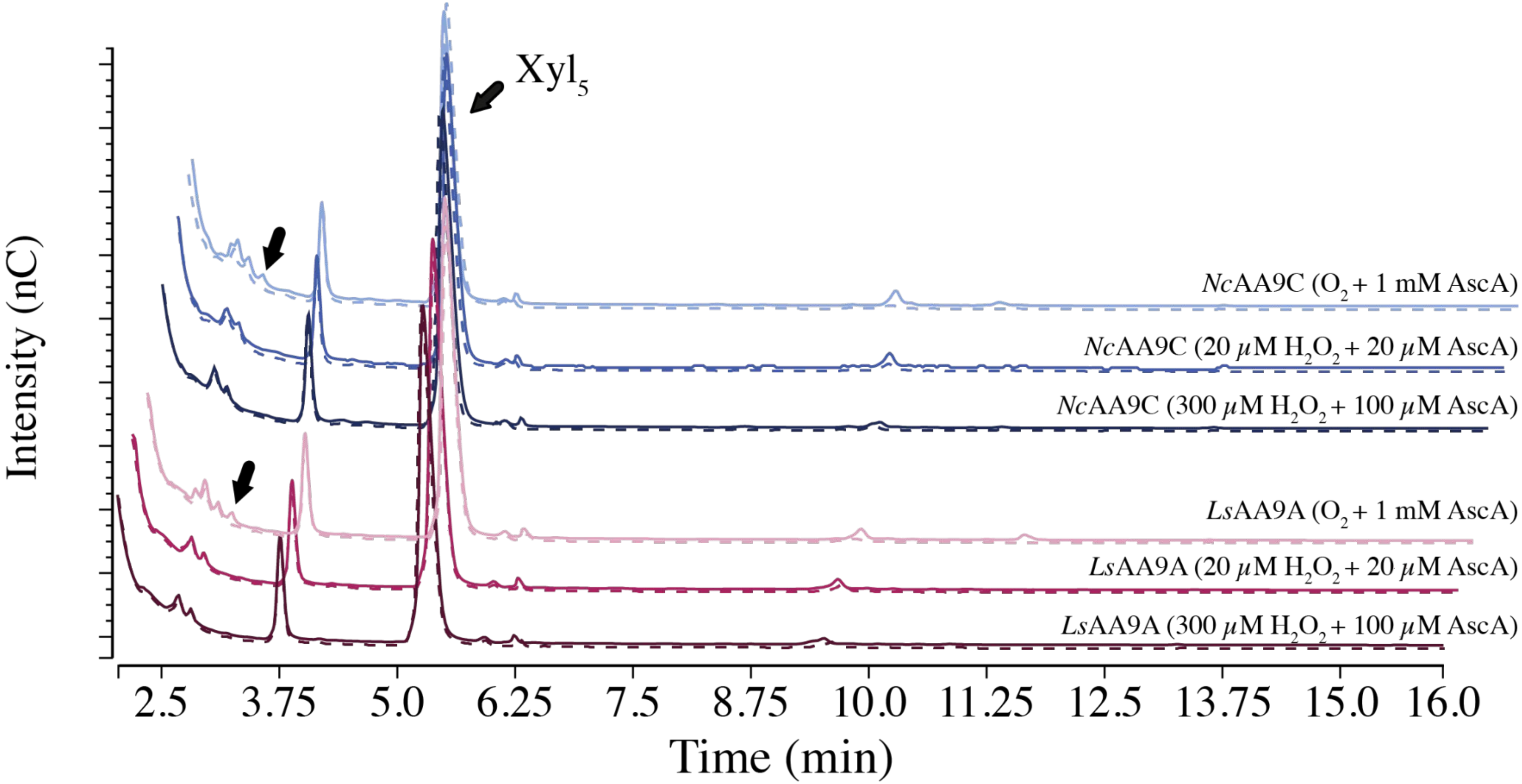

Figure S1. HPLC product profiles for reactions with 1 μM *Nc*AA9C (bluish colours) or *Ls*AA9A (purple colours) and 1 mM xylopentaose performed under standard aerobic conditions with the additions indicated in the chromatograms, and incubated overnight, at 37 ˚C. The dashed lines are chromatograms for

corresponding reactions without AscA. Unlabeled arrows indicate minor amounts of unidentified products that may derive form oxidative cleavage of xylopentaose.

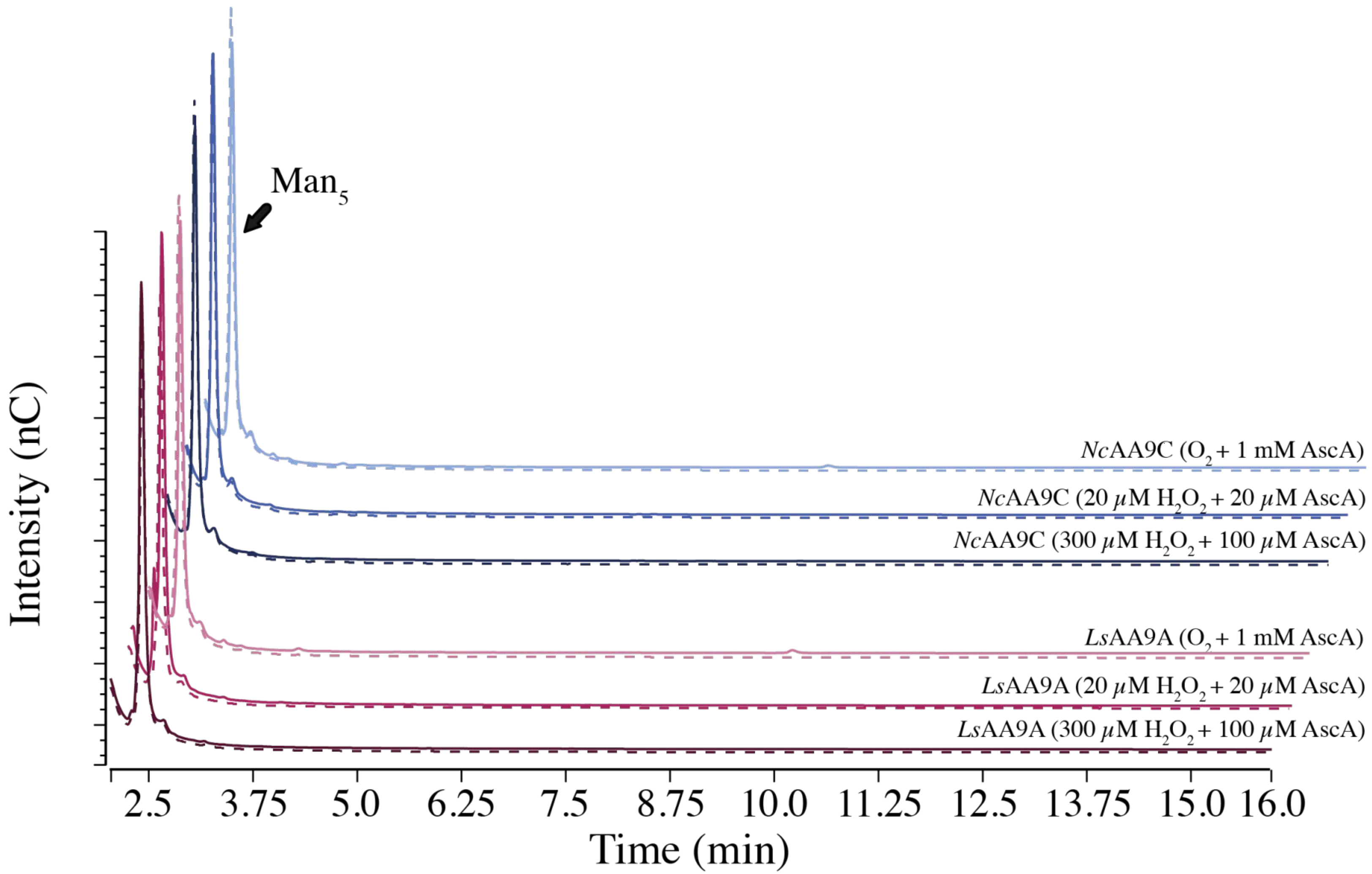

Figure S2. HPLC product profiles for reactions with 1 μM *Nc*AA9C (bluish colours) or *Ls*AA9A (purple colours) and 1 mM mannopentaose performed under standard aerobic conditions with the additions indicated in the chromatograms, and incubated overnight, at 37 ˚C. The dashed lines are chromatograms for corresponding reactions without AscA.

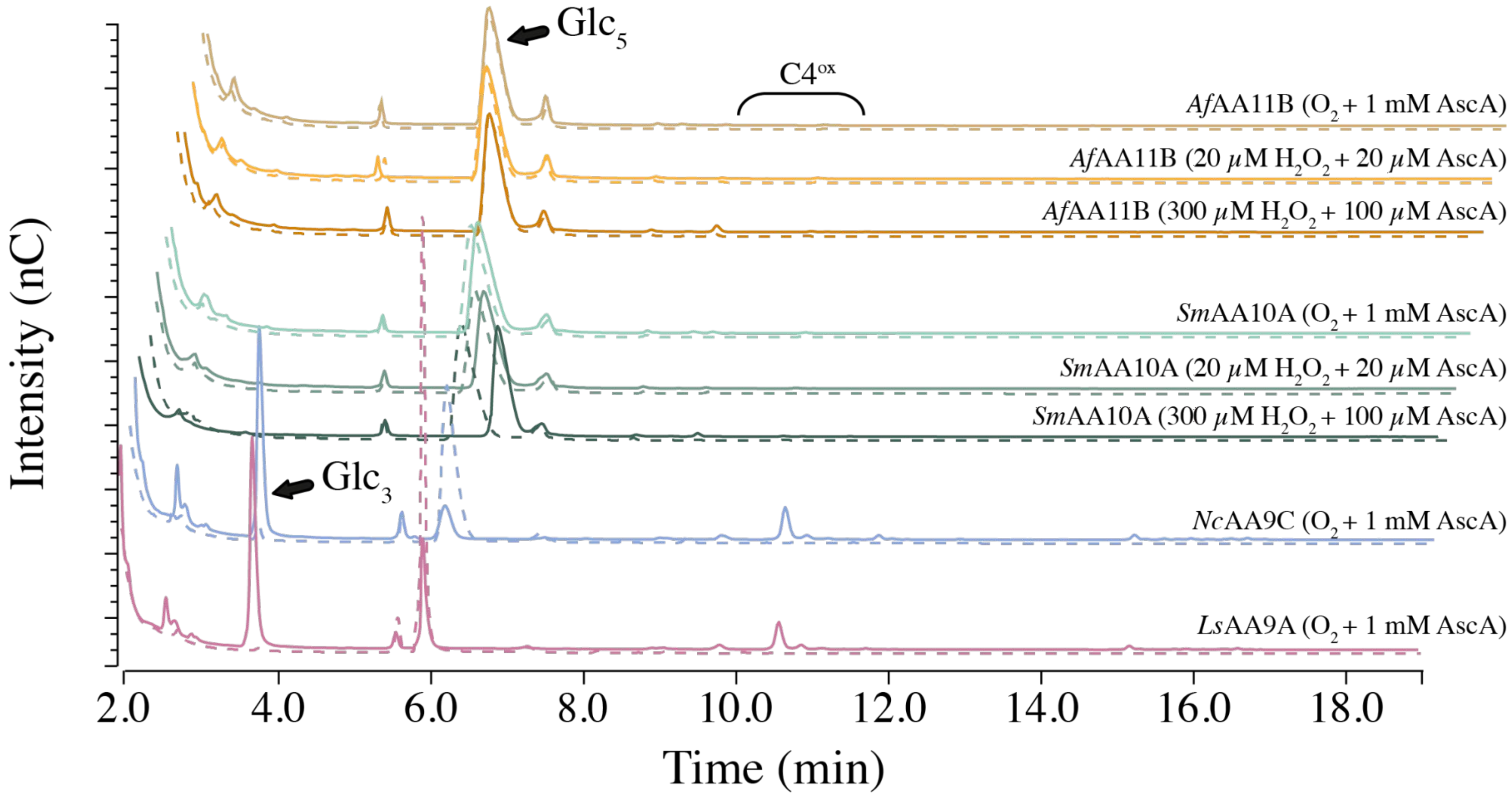

Figure S3. HPLC product profiles for reactions with 1 µM *Af*AA11B (yellow colours) or *Sm*AA10A (green colours) and 1 mM cellopentaose performed under standard aerobic conditions with the additions indicated in the chromatograms, and incubated overnight, at 37 ˚C. For comparison, positive controls

showing products generated upon aerobic overnight reactions with *Nc*AA9C (blue) and *Ls*AA9A (purple) are included. The position of peaks corresponding to C4-oxidized products is indicated by "C4^{ox}". The dashed lines are chromatograms for corresponding reactions without AscA.
